# Short- and Long-Term Outcomes After Hepatectomy in Patients Receiving Triplet Versus Doublet Preoperative Chemotherapy for Colorectal Liver Metastases

**DOI:** 10.1245/s10434-025-18413-3

**Published:** 2025-10-01

**Authors:** Agostino M. De Rose, Elena Panettieri, Mateo Lendoire, Yosuke Nakao, Eleanor A. Fallon, Caterina Mele, Marc-Antoine Allard, Timothy E. Newhook, Francesco Ardito, René Adam, Jean-Nicolas Vauthey, Felice Giuliante

**Affiliations:** 1 Hepatobiliary Surgery, Fondazione Policlinico Universitario Agostino Gemelli IRCCS, Rome, Italy; 2 Department of Surgical Oncology, The University of Texas MD Anderson Cancer Center, Houston, TX USA; 3 Hepatobiliary and Transplant Unit, AP-HP Hôpital Paul Brousse, Université Paris-Saclay, Villejuif, France

## Abstract

**Background:**

FOLFOXIRI is associated with better response and higher resection rates than doublet regimens for colorectal liver metastases (CLM); however, irinotecan and oxaliplatin may cause liver injury, increasing the risk of hepatectomy. This study compared outcomes between patients receiving FOLFOXIRI versus FOLFOX/FOLFIRI before undergoing CLM resection.

**Methods:**

This multicenter retrospective study included consecutive patients undergoing CLM resection during 2010–2021 after preoperative FOLFOXIRI or FOLFOX/FOLFIRI as first-line chemotherapy. Patients receiving multiple lines of therapy were excluded.

**Results:**

Of 1711 patients included, 160 (9.4%) received FOLFOXIRI and 1551 (90.6%) received FOLFOX/FOLFIRI. Patients receiving FOLFOXIRI were younger (median age 55 vs. 59 years; *p* < 0.001), had a higher rate of synchronous CLM (93.7% vs. 78.7%; *p* < 0.001), and had a higher median tumor burden score (TBS; 5.6 vs. 4.1; *p* < 0.001). In both groups, a median of six cycles of preoperative chemotherapy were administered. Outcomes after FOLFOXIRI (vs. FOLFOX/FOLFIRI) were as follows: major hepatectomy, 44.4% of patients (vs. 35.3%; *p* = 0.024), median estimated blood loss, 300 mL (vs. 200 mL; *p* = 0.006), rate of intraoperative blood transfusion, 8.1% (vs. 4.4%; *p* = 0.034), 90-day major complication rate, 13.3% (vs. 10.0%; *p* = 0.193), 90-day mortality rate, 1.2% (vs. 0.6%; *p* = 0.324), and 5-year overall survival (OS) rate, 66.6% (vs. 56.4%; *p* = 0.072). Medium–high TBS, synchronous extrahepatic disease, positive resection margin, and *RAS* mutation were independent risk factors for poor OS. Patients with medium–high TBS had a significantly better 5-year OS rate after FOLFOXIRI than after FOLFOX/FOLFIRI (67.6% vs. 50.1%; *p* = 0.004).

**Conclusion:**

In patients with CLM with higher disease burden, FOLFOXIRI may be preferable to FOLFOX/FOLFIRI for preoperative chemotherapy.

The standard treatment for colorectal liver metastases (CLM) is resection, and patients treated with resection may experience long-term disease-free survival or even cure. Improved surgical technique and better efficacy of chemotherapy have expanded the proportion of patients with CLM who are eligible for liver resection, and there is a strong correlation between response rates after chemotherapy and resection rates.^1^

For decades, 5-fluorouracil has been the backbone of systemic treatment for metastatic colorectal cancer (mCRC). Since the approval of irinotecan and oxaliplatin, 5-fluorouracil has generally been combined with one or both of these agents for the treatment of CLM; however, administration of regimens based on irinotecan and/or oxaliplatin can cause liver injury.^2,3^ Irinotecan-based regimens increase the risk of steatohepatitis, and oxaliplatin-based regimens increase the risk of sinusoidal obstruction,^4–6^ which translates into increased overall morbidity, mortality, and post-hepatectomy liver failure (PHLF) rates for irinotecan,^4^ and increased perioperative transfusion rate after hepatectomy for oxaliplatin.^7^ Furthermore, the risk of complications related to liver injury after hepatectomy increases with the number of cycles of chemotherapy administered, therefore the potential benefits of prolonged chemotherapy must be balanced against the risk of such complications.^6,8,9^

Several randomized trials have demonstrated that among patients with CLM, systemic treatment with 5-fluorouracil, leucovorin, folinic acid, oxaliplatin, and irinotecan (FOLFOXIRI), a so-called triplet regimen, is associated with higher response and resection rates than systemic treatment with 5-fluorouracil, leucovorin, folinic acid, and oxaliplatin (FOLFOX) or 5-fluorouracil, leucovorin, folinic acid, and irinotecan (FOLFIRI), so-called doublet regimens.^10–14^

The aim of this study was to compare perioperative outcomes and long-term survival between patients who received triplet chemotherapy and those who received doublet chemotherapy before liver resection for CLM.

## Methods

### Patient Selection

Data from all consecutive patients undergoing first curative-intent liver resection for CLM from January 2010 through December 2021 at three participating centers—Fondazione Policlinico Universitario Agostino Gemelli IRCCS (Rome, Italy), The University of Texas MD Anderson Cancer Center (Houston, TX, USA), and AP-HP Hôpital Paul Brousse, Université Paris-Saclay (Villejuif, France)—were retrospectively analyzed.

Patients at least 18 years of age who underwent first curative-intent hepatectomy after preoperative chemotherapy were identified. Patients who did not receive preoperative chemotherapy or who received preoperative chemotherapy with regimens other than FOLFOXIRI, FOLFOX, or FOLFIRI were excluded. Patients who underwent repeat hepatectomy were included once in the dataset, and only data for the index hepatectomy were taken into account. Each two-stage hepatectomy was recorded as a single liver resection.

### Ethics Statement

This study was approved by the Institutional Review Board of Fondazione Policlinico Universitario Agostino Gemelli IRCCS, Università Cattolica del Sacro Cuore, and subsequently approved by the Institutional Review Boards of the other two participating centers.

Data collection and analysis were performed according to institutional guidelines and conformed to the ethical standards of the World Medical Association (Declaration of Helsinki). Rigorously de-identified data were shared between institutions.

### Definitions

In this report, *doublet therapy* refers to FOLFOX or FOLFIRI and *triplet therapy* refers to FOLFOXIRI. Patients’ underlying physiologic status was assessed according to the American Society of Anesthesiologists (ASA) score.^15^ Synchronous CLMs were defined as metastases diagnosed within 12 months of primary tumor diagnosis.^16^ The tumor burden score (TBS) was calculated on the basis of tumor size and total number of tumors as follows: TBS^2^ = (maximum diameter)^2^ + (number of tumors)^2^.^17^ TBS values were categorized as low (<3), medium (≥3 to <9), and high (≥9) as originally proposed.^17^ Colorectal cancer staging was based on the Eighth Edition of the *American Joint Committee on Cancer Staging Manual*.^18^ Type of liver resection (major vs. minor) was based on the Brisbane classification.^19^ Sequencing of primary tumor resection and CLM resection was classified as simultaneous, primary tumor resection first (the classic approach), or CLM resection first (liver-first approach).^20^ The estimated blood loss was independently assessed at each participating institution and measured in milliliters (mL). R1 resection was defined as a parenchymal tumor-free margin <1 mm^21^ or as CLM detachment from first/second-order glissonean pedicles or from hepatic veins within their last 4 cm before confluence into the inferior vena cava.^22^ Complications were described according to the Clavien–Dindo classification.^23^ Major morbidity refers to Clavien–Dindo grade 3 or greater complications. PHLF^24^ and bile leak^25^ were defined according to the International Study Group for Liver Surgery.

Overall survival (OS) was defined as months between surgical resection and death.

### Preoperative Assessment

At all participating centers, there were no predefined criteria of unresectability with regard to number, size, and CLM laterality. Unresectability was defined by inadequate future liver remnant or by the impossibility to remove all CLMs either by a one- or two-stage procedure. An anticipated risk of R1 resection was not a contraindication to liver resection, although the strategy was to obtain a tumor-free margin, whenever possible.

### Statistical Analysis

Quantitative variables were reported as median and interquartile range (IQR), and qualitative variables were reported as absolute and relative frequency (percentage). Cut-offs for individual variables were selected on the basis of the median value in the entire cohort.

Quantitative variables were compared using the Mann–Whitney U test, while categorical variables were compared using the chi-square test.

Simple and multiple logistic regression models were performed to predict the likelihood of development of major postoperative complications. Survival curves were generated using the Kaplan–Meier method, and the differences between the curves were assessed using the log-rank test. Univariable and multivariable analyses to identify factors associated with OS were performed using Cox proportional hazards regression models. A subgroup survival analysis was performed for patients with medium–high TBS.

All statistical tests were two-sided and statistical significance was defined as *p <* 0.05. Analyses were performed using STATA software (version 14.0; StataCorp LLC, College Station, TX, USA).

## Results

### Study Population

Of 1711 patients included, 160 (9.3%) received preoperative chemotherapy with the triplet regimen, and 1551 (90.6%) received preoperative chemotherapy with a doublet regimen. Compared with the patients treated with doublet therapy, the patients treated with FOLFOXIRI were younger (median age 55 vs. 59 years; *p* < 0.001), less frequently had a body mass index of 30 kg/m^2^ or greater (16.0% vs. 28.7%; *p* < 0.001), and less frequently had an ASA score of III or greater (37.6 vs. 61.2 %; *p* < 0.001).

**Table 1:** Patient and tumor characteristics of patients who underwent resection of colorectal liver metastases after triplet or doublet preoperative chemotherapy

	Entire population	Triplet regimen	Doublet regimen	*p*-value
	[*n* = 1711]	[*n* = 160]	[*n* = 1551]	
*Demographic factors*				
Age, years [median (IQR)]	58 (49–66)	55 (46–64)	59 (50–67)	*<0.001*
Males	1038 (60.7)	94 (58.7)	944 (60.9)	0.595
BMI ≥30 kg/m^2a^	413 (27.5)	23 (16.0)	390 (28.7)	*<0.001*
ASA score ≥III^b^	578 (58.1)	50 (37.6)	528 (61.2)	*<0.001*
*CLM factors*				
Synchronous^c^	1356 (80.1)	150 (93.7)	1206 (78.7)^c^	*<0.001*
Bilateral^d^	617 (64.1)	106 (66.2)	511 (64.1) ^d^	0.529
Number of CLMs [median (IQR)]^e^	2 (1–5)	4 (2–7)	2 (1–4)	*<0.001*
CLM maximum size, cm [median (IQR)]^f^	2.5 (1.5–4)	2.7 (1.6–4.8)	2.5 (1.5–3.8)	*0.015*
TBS [median (IQR)]^g^	4.2 (2.7–6.7)	5.6 (3.6–9.4)	4.1 (2.7–6.5)	*<0.001*
TBS grade^g^				
Low	483 (28.8)	23 (14.9)	460 (30.3)	*<0.001*
Medium	976 (58.3)	88 (57.1)	888 (58.4)	*–*
High	215 (12.8)	43 (27.9)	172 (11.3)	*–*
Synchronous extrahepatic disease^h^	315 (18.8)	38 (23.9)	277 (18.3)	0.085
*Primary tumor factors*				
Rectal cancer^i^	498 (29.2)	53 (33.3)	445 (28.8)	0.23
Right colon cancer^i^	394 (23.1)	39 (24.5)	355 (23.0)	0.655
T category ≥3^j^	591 (88.9)	137 (92.6)	454 (87.1)	0.105
Positive lymph nodes^k^	458 (50.0)	114 (77.5)	344 (44.8)	*<0.001*
*Chemotherapy factors*				
Number of cycles [median (IQR)]^l^	6 (4–8)	6 (5–9)	6 (4–8)	0.759
Targeted therapies	1289 (75.3)	120 (75.0)	1169 (75.4)	0.917
Bevacizumab^m^	1003 (58.7)	91 (56.9)	912 (58.9)	0.624
*RAS mutation*^**n**^	659 (47.4)	42 (46.7)	617 (47.4)	0.889

Data are expressed as *n* (%) unless otherwise specified

*IQR* interquartile range, *BMI* body mass index, *ASA* American Society of Anesthesiologists, *CLM* colorectal liver metastases, *TBS* tumor burden score

^a^ Data missing on BMI for 207 patients

^b^ Data missing on ASA score for 716 patients

^c^ Data missing on timing of CLM diagnosis for 18 patients

^d^ Data missing on CLM laterality for 748 patients

^e^ Data missing on number of CLM for 10 patients

^f^ Data missing on CLM maximum size for 24 patients

^g^ Data missing on TBS for 37 patients

^h^ Data missing on synchronous extrahepatic disease for 37 patients

^i^ Data missing on primary tumor location for 6 patients

^j^ Data missing on T category for 1046 patients

^k^ Data missing on N category for 796 patients

^l^ Data missing on number of chemotherapy cycles for 34 patients

^m^ Data missing on type of targeted therapy for 2 patients

^n^ Data missing on *RAS* mutational status for 320 patients

Compared with patients treated with doublet regimens, patients treated with FOLFOXIRI had a higher rate of synchronous CLM (93.7% vs. 78.7%; *p* < 0.001), had more CLM (median 4 vs. 2; *p* < 0.001), had bigger CLM (median 2.7 vs. 2.5 cm; *p* = 0.015), and had a higher median TBS (5.6 vs. 4.1; *p* < 0.001). In both groups, a median of six cycles of preoperative chemotherapy were administered. The rate of administration of targeted therapies was similar in the FOLFOXIRI and doublet-regimen groups (75.0% and 75.4%, respectively; *p* = 0.917) (Table 1).

### Intraoperative Outcomes

Intraoperative outcomes in the FOLFOXIRI and doublet-regimen groups, respectively, were as follows: rate of major hepatectomy, 44.4% and 35.3% (*p* = 0.024); rate of a liver-first approach, 28.7% and 15.0% (*p* < 0.001); median estimated blood loss, 300 mL and 200 mL (*p* = 0.006); rate of intraoperative blood transfusion, 8.1% and 4.4% (*p* = 0.034); and median operative time, 400 minutes and 300 minutes (*p* < 0.001) (Table 2).

**Table 2:** Intraoperative and short-term postoperative outcomes of patients who underwent resection of colorectal liver metastases after triplet or doublet preoperative chemotherapy

	Entire population	Triplet regimen	Doublet regimen	*p*-value
	[*n* = 1711]	[*n* = 160]	[*n* = 1551]	
*Intraoperative outcomes*				
Major hepatectomy	619 (36.2)	71 (44.4)	548 (35.3)	*0.024*
Liver-first approach^a^	274 (16.3)	46 (28.7)	228 (15.0)	*<0.001*
Simultaneous colorectal resection^a^	209 (12.4)	19 (11.9)	190 (12.5)	0.829
EBL, mL [median (IQR)]^b^	200 (100–400)	300 (150–500)	200 (100–400)	*0.006*
EBL >200 mL^b^	570 (47.6)	54 (55.7)	516 (46.9)	0.099
Intraoperative transfusion^c^	81 (4.7)	13 (8.1)	68 (4.4)	*0.034*
Operative time, min [median (IQR)]^d^	307 (233–402)	400 (310–516)	300 (225–393)	*<0.001*
Operative time >300 min^d^	827 (51.8)	96 (76.8)	731 (49.7)	*<0.001*
R1 resection^e^	406 (23.8)	118 (73.7)	1183 (76.5)	0.442
*Short-term outcomes*				
90-day mortality	11 (0.6)	2 (1.2)	9 (0.6)	0.324
90-day major complication^f^	175 (10.3)	21 (13.3)	154 (10.0)	0.193
Bile leak	87 (5.1)	16 (10.0)	71 (4.6)	*0.003*
Grade A	18 (1.1)	3 (1.9)	15 (1.0)	0.284
Grade B	66 (3.9)	11 (6.9)	55 (3.5)	*0.037*
Grade C	3 (0.2)	2 (1.2)	1 (0.06)	*<0.001*
PHLF	100 (5.8)	6 (3.7)	94 (6.1)	0.235
Grade A	51 (3.0)	5 (3.1)	46 (3.0)	0.91
Grade B	42 (2.5)	1 (0.6)	41 (2.6)	0.116
Grade C	7 (0.4)	0 (0)	7 (0.4)	0.394

Data are expressed as *n* (%) unless otherwise specified

*EBL* estimated blood loss, *IQR* interquartile range, *PHLF* post-hepatectomy liver failure

^a^ Data missing on sequencing of primary tumor resection and CLM resection for 27 patients

^b^ Data missing on EBL for 514 patients

^c^ Data missing on intraoperative transfusions for 2 patients

^d^ Data missing on operative time for 114 patients

^e^ Data missing on margin status for 4 patients

^f^ Excluding postoperative deaths

### Short-Term Outcomes

In the FOLFOXIRI and doublet-regimen groups, respectively, the mortality rates at 90 days were 1.2% and 0.6% (*p* = 0.324), and the major complication rates at 90 days were 13.3% and 10.0% (*p* = 0.193). Of the two patients treated with preoperative FOLFOXIRI who died within 90 days after surgery, one developed grade B PHLF, followed by atrial fibrillation and heart failure, and the other had a grade C bile leak with associated abdominal collection, sepsis, and coronavirus disease 2019 (COVID-19) infection. Of the nine patients treated with doublet regimens who died within 90 days after surgery, six had grade C PHLF; two died from sepsis after bowel perforation and hemorrhagic shock, respectively; and one had a fatal arrhythmia.

With regard to liver-specific complications, postoperative bile leak was more common after treatment with FOLFOXIRI than after treatment with a doublet regimen (10.0% vs. 4.6%, *p* = 0.003), with significant differences in rates of grade B leaks (6.9% vs. 3.5%, *p =* 0.037) and grade C leaks (1.2% vs. 0.06%, *p* < 0.001). On the other hand, rates of PHLF were similar in the FOLFOXIRI and doublet-regimen groups (3.7% and 6.1%, respectively, *p* = 0.235) (Table 2). No center effect was observed in terms of major complications (*p* = 0.422) and 90-day mortality (*p =* 0.671).

Univariable and multivariable logistic regression models showed that number of preoperative chemotherapy cycles >6, simultaneous colorectal resection, major hepatectomy, and operative time >300 minutes were independently associated with major morbidity (Table 3).

**Table 3:** Univariable and multivariable logistic regression model for Clavien–Dindo complication grade 3 or higher in patients who underwent resection of colorectal liver metastases after triplet or doublet preoperative chemotherapy

Factor	No. of patients	No. of events	Univariable analysis			Multivariable analysis		
			OR	95% CI	*p*-value	HR	95% CI	*p*-value
*Patient factors*								
Age >60 years	748	82	1.02	0.75–1.38	0.914			
BMI ≥30	413	45	0.95	0.66–1.37	0.797			
ASA score III or higher	578	54	0.72	0.48–1.08	0.116			
*Preoperative factors*								
Triplet chemotherapy	160	23	1.43	0.89–2.29	0.137			
Chemotherapy >6 cycles	656	94	1.71	1.26–2.33	*0.001*	1.66	1.20–2.30	*0.002*
Bevacizumab	1003	109	0.99	0.73–1.36	0.980			
*Operative factors*								
Simultaneous colorectal resection	209	33	1.66	1.10–2.49	*0.015*	1.74	1.11–2.72	*0.016*
Major hepatectomy	619	93	1.90	1.40–2.58	*<0.001*	1.88	1.36–2.60	*<0.001*
Intraoperative blood transfusion	81	15	1.94	1.08–3.47	*0.026*	1.20	0.62–2.32	0.581
Operative time >300 min	827	114	1.73	1.26–2.39	*0.001*	1.53	1.09–2.15	*0.013*

*OR* odds ratio, *CI* confidence interval, *HR* hazard ratio, *BMI* body mass index, *ASA* American Society of Anesthesiologists

### Long-Term Outcomes

**Fig. 1:**
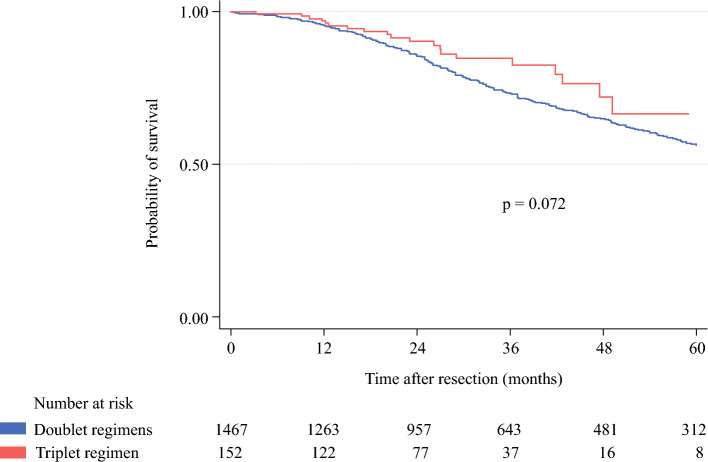
Overall survival of patients who underwent liver resection for colorectal liver metastases, stratified by type of preoperative chemotherapy. 1-, 3-, and 5-year overall survival rates were 97.0%, 84.7%, and 66.6%, respectively, for patients treated with the triplet regimen, and 95.4%, 73.3%, and 56.4%, respectively, for patients treated with a doublet regimen. Median overall survival was not reached for patients treated with the triplet regimen and 75.8 months for patients treated with a doublet regimen

Follow-up was available for 1619 patients. After a mean follow-up time of 39.0 months, the 1-, 3-, and 5 years OS rates were 97.0%, 84.7%, and 66.6%, respectively, for patients treated with FOLFOXIRI, and 95.4%, 73.3%, and 56.4%, respectively, for patients treated with a doublet regimen (*p* = 0.072). Median OS was 75.8 months for patients treated with FOLFOXIRI and was not reached for patients treated with a doublet regimen (Fig. 1). For the entire cohort, a medium–high TBS, synchronous extrahepatic disease, a positive (R1) resection margin, and *RAS* mutation were independent risk factors for poor OS (Table 4).

**Table 4:** Multivariable Cox proportional hazards model analysis for overall survival for patients who underwent resection of colorectal liver metastases after triplet or doublet preoperative chemotherapy

Factor	No. of patients	No. of events	Univariable analysis			Multivariable analysis		
			HR	95% CI	*p*-value	HR	95% CI	*p*-value
*Preoperative factors*								
Synchronous CLM	1301	407	1.05	0.84–1.32	0.649			
Medium–high TBS	1142	401	2.15	1.72–2.69	*<0.001*	2.05	1.58–2.67	*<0.001*
Synchronous extrahepatic disease	310	115	1.62	1.32–2.00	*<0.001*	1.55	1.24–1.94	*<0.001*
Triplet chemotherapy	153	21	0.67	0.43–1.04	0.074			
*Operative factors*								
Major hepatectomy	604	231	1.45	1.22–1.73	*<0.001*	1.16	0.95–1.42	0.153
R1 resection	386	135	1.58	1.30–1.93	*<0.001*	1.47	1.19–1.82	*<0.001*
RAS mutation	637	215	1.23	1.01–1.49	*0.036*	1.33	1.10–1.62	*0.004*

*HR* hazard ratio, *CI* confidence interval, *CLM* colorectal liver metastases, *TBS* tumor burden score

**Fig. 2:**
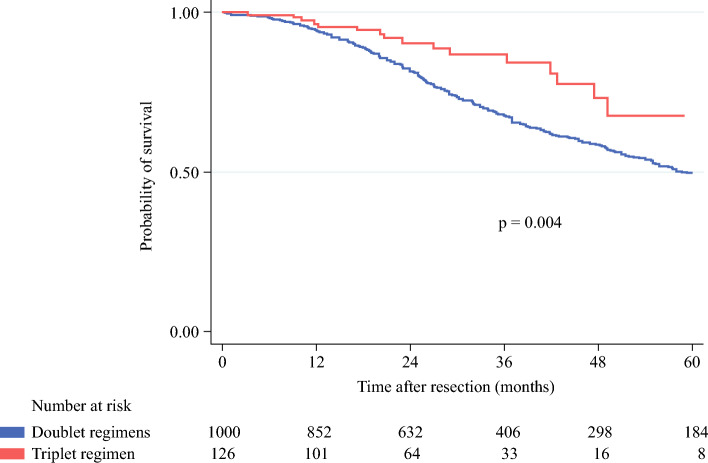
Overall survival of patients with a medium–high tumor burden score who underwent liver resection for colorectal liver metastases, stratified by type of preoperative chemotherapy. 1-, 3-, and 5-year overall survival rates were 96.4%, 87.0%, and 67.6%, respectively, for patients treated with the triplet regimen, and 94.3%, 68.1%, and 50.1%, respectively, for patients treated with a doublet regimen. Median overall survival was not reached for patients treated with the triplet regimen and 60.3 months for patients treated with a doublet regimen

In subgroup survival analysis of patients with medium–high TBS, patients who received FOLFOXIRI had a significantly higher 5-year OS rate than patients who received a doublet regimen (67.6% vs. 50.1%, *p* = 0.004) [Fig. 2]. Univariable and multivariable analyses confirmed that synchronous extrahepatic disease, a positive (R1) resection margin, and *RAS* mutation were independent risk factors for poor OS. Additionally, having received FOLFOXIRI was independently associated with improved OS (*p* = 0.038) [Table 5].

**Table 5:** Multivariable Cox proportional hazards model analysis for overall survival for patients with a medium–high tumor burden score who underwent resection of colorectal liver metastases after triplet or doublet preoperative chemotherapy

Factor	No. of patients	No. of events	Univariable analysis			Multivariable analysis		
			HR	95% CI	*p*-value	HR	95% CI	*p*-value
*Preoperative factors*								
Synchronous CLM	922	327	0.89	0.69–1.15	0.379			
Synchronous extrahepatic disease	215	94	1.71	1.35–2.15	*<0.001*	1.67	1.30–2.15	*<0.001*
Triplet chemotherapy	126	17	0.50	0.31–0.82	*0.006*	0.56	0.33–0.97	*0.038*
*Operative factors*								
Major hepatectomy	530	210	1.16	0.96–1.41	0.129			
R1 resection	318	120	1.41	1.14–1.75	*0.002*	1.40	1.11–1.76	*0.004*
*RAS mutation*	432	169	1.32	1.06–1.63	*0.012*	1.37	1.10–1.70	*0.004*

*HR* hazard ratio, *CI* confidence interval, *CLM* colorectal liver metastases

## Discussion

This study compared outcomes of patients undergoing hepatectomy for CLM following the administration of triplet versus doublet chemotherapy. Perioperative morbidity and mortality rates were similar for CLM resection after preoperative FOLFOXIRI and CLM resection after preoperative doublet therapy. Despite having more advanced disease, patients treated with triplet chemotherapy had OS similar to that of patients treated with doublet regimens. Notably, in patients with a higher disease burden, FOLFOXIRI appeared to be associated with improved survival.

Over the past decade, advances in understanding of both patient- and tumor-specific prognostic factors, together with increased availability of effective treatment options, have greatly shaped the management of mCRC. Several phase II and III randomized clinical trials comparing FOLFOXIRI with doublet regimens (FOLFOX and FOLFIRI) demonstrated that intensifying upfront chemotherapy provided benefits for patients with mCRC.^10–14^ Addition of bevacizumab to FOLFOXIRI has also become standard. Notably, a meta-analysis of five clinical trials confirmed that FOLFOXIRI plus bevacizumab (*n* = 846) significantly improved the progression-free survival, objective response rate, and R0 resection rate of patients with mCRC compared with a doublet regimen plus bevacizumab (*n* = 851).^26^ Given this finding, it is unsurprising that FOLFOXIRI plus bevacizumab has been incorporated among the first-line treatment options for mCRC in most clinical guidelines and recommendations worldwide.^27–30^

Although FOLFOXIRI for CLM has been studied extensively, all the available studies focused on the safety and efficacy of the triplet regimen, reporting response to treatment, resection rate, and surgical radicality but not postoperative outcomes and long-term survival. While the benefits of downsizing tumors through aggressive chemotherapy are well recognized, there has been a lack of evidence regarding operative risk of liver resection in these patients. Assessing the impact of postoperative complications on survival appears particularly important as a recent systematic review and meta-analysis that included 12,817 patients from 41 studies highlighted a significant negative effect of postoperative complications on both long-term survival and recurrence rates in patients undergoing resection for CLM.^31^

A recent analysis of electronic health records of 36,056 patients treated for mCRC between 2010 and 2021 showed that triplet chemotherapy regimens were more likely to be used in younger patients, particularly in the first-line setting.^32^ This is confirmed by the results in the present paper. We found that patients who underwent resection following FOLFOXIRI were in fact significantly younger, had fewer comorbidities, and had a higher liver tumor burden than patients treated with doublet regimens. Moreover, notably higher percentages of the patients treated with FOLFOXIRI than of those treated with doublet regimens had synchronous CLM and required a liver-first approach. Further evidence of more advanced disease in the FOLFOXIRI group was their higher rate of major hepatectomy, longer operative time, and higher rate of intraoperative blood transfusion.

Despite these contrasts, no significant difference was found between the FOLFOXIRI and doublet-regimen groups in terms of 90-day mortality or 90-day major complications. Most important, no difference was found between the groups in the incidence of PHLF, which is a crucial outcome in liver surgery. There was, however, a significantly higher incidence of postoperative bile leak in patients who received preoperative FOLFOXIRI, a finding that requires further investigation to confirm or rule out a causal relationship. Notably, while no center effect on 90-day mortality or 90-day major complications was identified, it is important to highlight that differences in short-term outcomes may reflect the type of surgery performed and technical attitudes of the different surgeons/centers involved. The results of this study support the safety of liver resection after administration of FOLFOXIRI, since surgical risk was not increased compared with the risk after doublet regimens, and was in line with the risk reported in previous studies.^33,34^ The multivariable analysis showed that simultaneous colorectal resection, major hepatectomy, longer operative time, and more than six cycles of preoperative chemotherapy were the main independent predictors of major postoperative complications. Regarding the surgical factors related to major complications, the gold standard for treating CLM has evolved from traditional anatomic resection to parenchyma-sparing hepatectomy.^35,36^ The parenchyma-sparing approach prioritizes preserving as much healthy liver tissue as possible while achieving clear surgical margins, which is particularly important for patients with multiple CLM or patients who may require repeat resections. Additionally, simultaneous resection of the primary tumor and CLM is increasingly seen as an attractive alternative to the traditional staged approach. However, the decision to perform simultaneous colorectal and liver resections must be individualized, taking into account several patient- and disease-related factors, such as the patient’s general condition, the tumor burden, and the complexity of the required liver and colorectal procedures.^37^ Balancing risks and benefits is crucial to optimize outcomes, especially in cases of advanced or synchronous disease. The multivariable analysis also revealed that longer preoperative chemotherapy duration (>6 cycles) was significantly associated with an increased risk of major postoperative complications. The optimal duration of chemotherapy before surgical resection for CLM remains uncertain. Across various studies examining perioperative chemotherapy for CLM, the number of neoadjuvant chemotherapy cycles varied and most trials suggested the administration of 6–10 preoperative cycles.^38^ However, it is crucial to avoid unnecessarily prolonged systemic therapy before planned hepatectomy. Even in patients with initially unresectable disease, a radiographic assessment should be conducted every 2–3 months from the start of treatment, and surgery should be performed as soon as resectability is achieved to reduce the risk of postoperative complications. This approach prioritizes minimizing exposure to prolonged chemotherapy while optimizing surgical outcomes.

Notably, the survival analysis demonstrated better survival, although the difference was not statistically significant, in the patients who received neoadjuvant chemotherapy with FOLFOXIRI than in the patients who received neoadjuvant chemotherapy with doublet regimens. This improvement was observed even though the patients treated with FOLFOXIRI had unfavorable prognostic characteristics, including higher rates of synchronous CLM and a higher median TBS. These results require further investigation through comparative analysis, particularly in groups of patients matched for clinical and mutational characteristics, in order to identify a subpopulation that might benefit the most from neoadjuvant treatment with the triplet regimen. Nonetheless, even after various biases are taken into account, the median survival of approximately 76 months in the patients treated with FOLFOXIRI and their 5-year OS rate of 66% are very encouraging. In contrast with what was observed in the entire population, analysis of the subgroup of patients with a medium–high TBS showed that the triplet regimen was associated with an improved prognosis. This confirms that patients with a higher disease burden benefit the most from treatment with FOLFOXIRI. In this scenario, it is important to remember that FOLFOX still represents the most commonly administered preoperative chemotherapy regimen, due to its more favorable toxicity profile, and that selecting FOLFOXIRI as first-line treatment may interfere with treatment choices in the adjuvant setting or in case or recurrence.^28,39^

This study has several limitations. First, its retrospective design may have introduced unmeasured confounding. Reliable information on the initial indication for chemotherapy was unavailable, and the proportion of patients who were initially unresectable could not be determined. Furthermore, no balancing or matching analyses were conducted because triplet and doublet regimens are not clinically interchangeable, thereby violating the positivity (overlap) assumption and resulting in extreme weights and unstable estimates. Positivity implies that each individual has a non-zero probability of receiving any treatment, thereby approximating the conditions of a randomized trial. In this study, the violation of this assumption, together with the marked group imbalance and non-negligible missing data on key covariates, compromised the feasibility and reliability of matching approaches.

To the knowledge of the authors, this is the first study to investigate intraoperative and postoperative outcomes of patients receiving triplet preoperative chemotherapy for CLM and compare outcomes between these patients and those who received doublet chemotherapy over the past decade. The study’s demonstration that triplet chemotherapy does not significantly increase the operative risk and represents a safe therapeutic option for patients with advanced disease proves that an aggressive preoperative approach does not preclude curative-intent surgery and prolonged survival.

## Data Availability

The authors have not planned to make their data available to other researchers and did not preregister the research in an independent, institutional registry.
